# Supplementary material for: Analytical protocol for measuring micro-molar quantities of sulfur volatile species in experimental high pressure and temperature fluids
Source: Commun Chem. 2025 Jan 23;8:19. doi: 10.1038/s42004-024-01370-5 (PMC11758018; doi:10.1038/s42004-024-01370-5)
Supplement: Supplementary file 2 — Supplemental Information [file 42004_2024_1370_MOESM2_ESM.pdf]

Supplementary Material: Analytical protocol for measuring micro-molar quantities of sulfur volatile species in experimental high pressure and temperature fluids

Arianna Secchiari[1], Sandro Recchia[2], Luca Toffolo[1], Simone Tumiati[1]

[1] Dipartimento di Scienze Della Terra, Università Degli Studi di Milano, via Mangiagalli 34, I-20133 Milano, Italy.
[2] Dipartimento di Scienza e Alta Tecnologia, Università degli Studi dell'Insubria, via Valleggio 11, I-22100 Como, Italy.

Supplementary Figure 1: Back-scattered electron images of solid run products and compositional X-rays maps (grey scale) of sulfur and iron for the performed experiments. Solid run products reported as follows: a) SOH-IW2; d) SOH-IW1; g) SOH-FMQ1. Compositional X-rays maps of sulfur are illustrated in b) SOH-IW2; e) SOH-IW1; h) SOH-FMQ1. Compositional X-rays maps of iron are show in c) SOH-IW2; f) SOH-IW1; i) SOH-FMQ1.

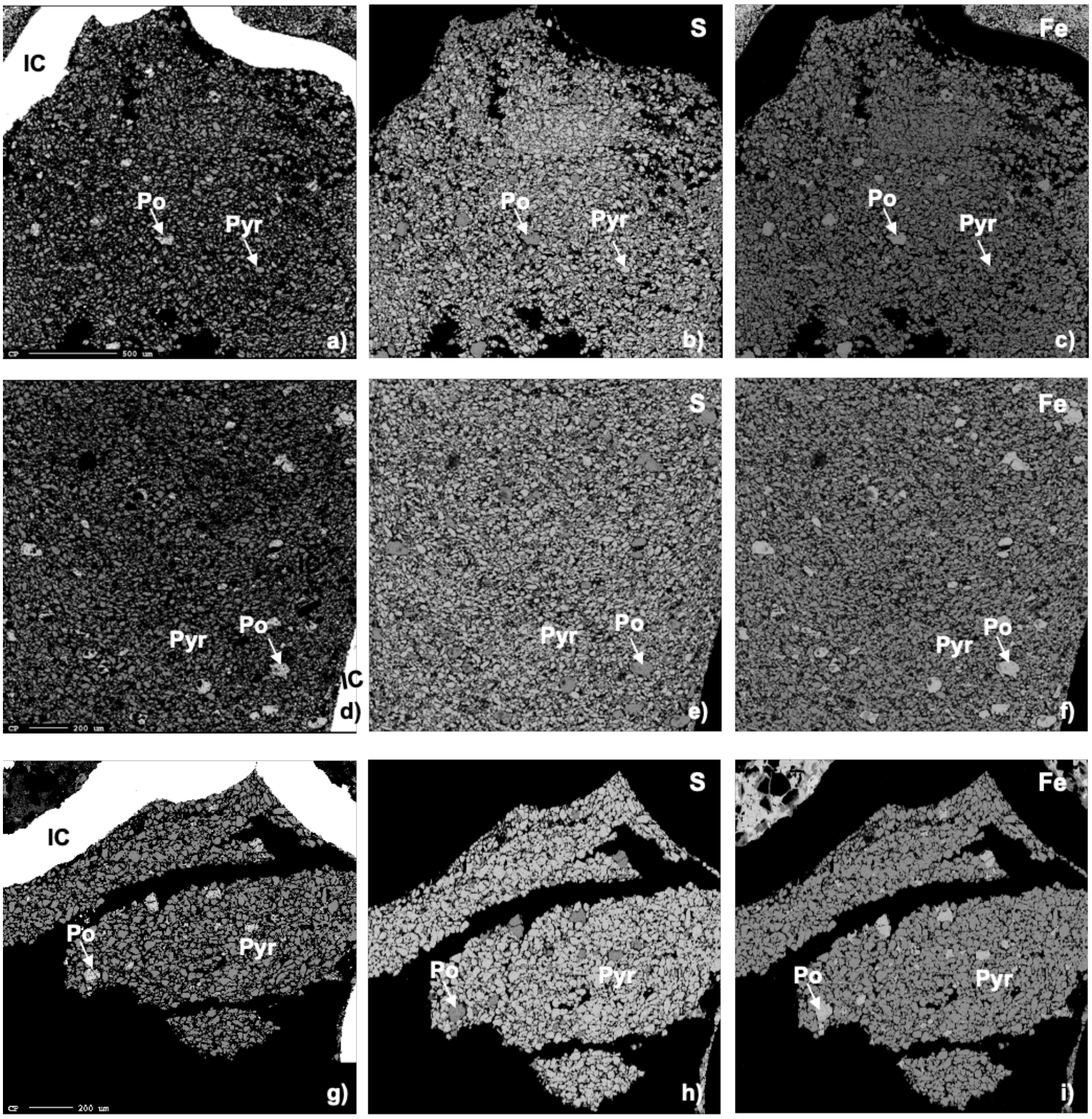

Supplementary Figure 2: diagram reporting *m/z* signal integration (peak area) for channel 34 (H_2S) measured during run SOH-IW1.

Reported on x axis = time, on y axis = signal (a.u.)

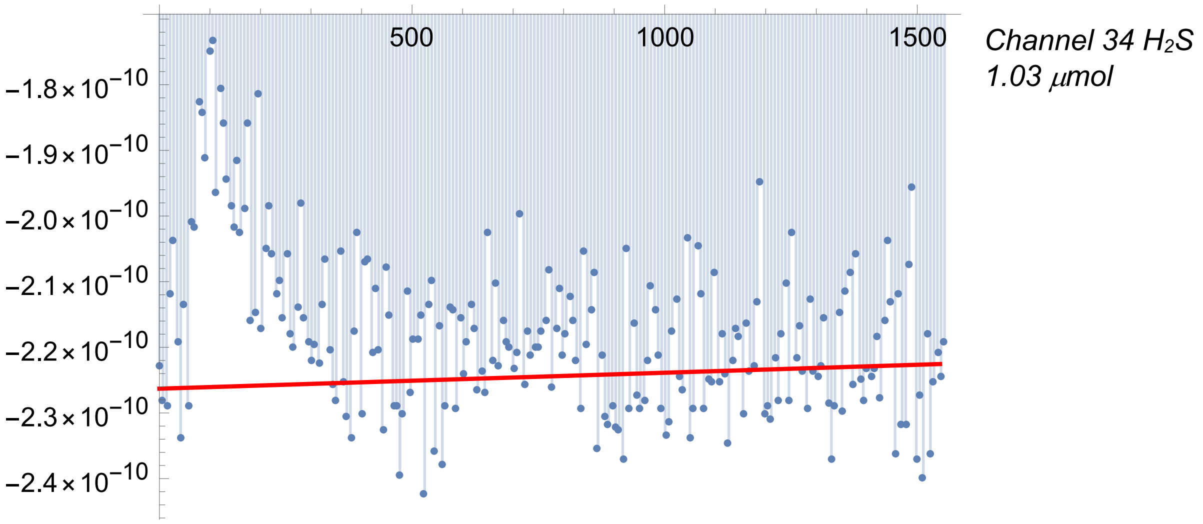

Supplementary Results

Capsule weakening effect related to H_2S formation

In addition to the all the experiments performed for this work (Supplementary Table 1), a series of three additional experiments (Supplementary Table 2) was conducted to evaluate the potential impact of temperature and pressure on capsule weakening. In each of these experiments, the runtime was consistently set to five hours, while temperature and pressure were systematically varied to assess their effects on capsule resistance. The chosen temperature and pressure conditions were based on subduction zones thermal models[1].

Given that our protocol is specifically designed to investigate fluid-solid equilibria, we aimed to maintain the temperature confidently below the melting temperature of the sulfide phase, which has been constrained at approximately 900°C for a pressure of 3 GPa[2].

Experiments performed at 3 GPa with variable temperatures of 800°C (SOH-IW3) and 600°C (SOH-IW4) highlighted the importance of kinetics in governing the H_2S-forming reaction, as indicated by fluid analysis.

In run SOH-IW3 (T= 800°C), the capsule was found empty (Supplementary Table 2), attesting that capsule weakening occurred within five hours, favoured by the elevated temperature. In contrast, run SOH-IW4 (T= 600°C) yielded a fluid entirely composed of H_2O (Supplementary Table 2 and Supplementary Fig. 3a), suggesting that the H_2S-forming reaction was inhibited at these lower temperatures (Supplementary Fig. 3b). Pressure variations, however, do not appear to significantly influence the capsule resistance. In run SOH-IW5 (Supplementary Fig. 4a, b, c, d), conducted at P= 1 GPa and T= 700°C, we recovered a fluid composed of 78.8 mol% H_2O, 18.9 mol% H_2S, 7.3 mol% SO_2, and minor amounts of H_2 (1.8 mol%), resulting in a ΔP of 102 mbar corresponding to 101.84 mmol.

These findings indicate that the established protocol, developed for a fixed pressure and temperature, can be effectively extended to varying temperature and pressure conditions. However, experiments SOH-IW3 and SOH-IW4 call for a more comprehensive investigation of the studied system to adequately constrain an optimized runtime. This task lies beyond the scope of the presented study and will be further addressed in future works.

Supplementary Figure 3: diagram reporting *m/z* signal integration (peak area) for **a)** channel 18 (H_2O) and **b)** channel 34 (H_2S) measured during run SOH-IW4.

Reported on x axis = time, on y axis = signal (a.u.)

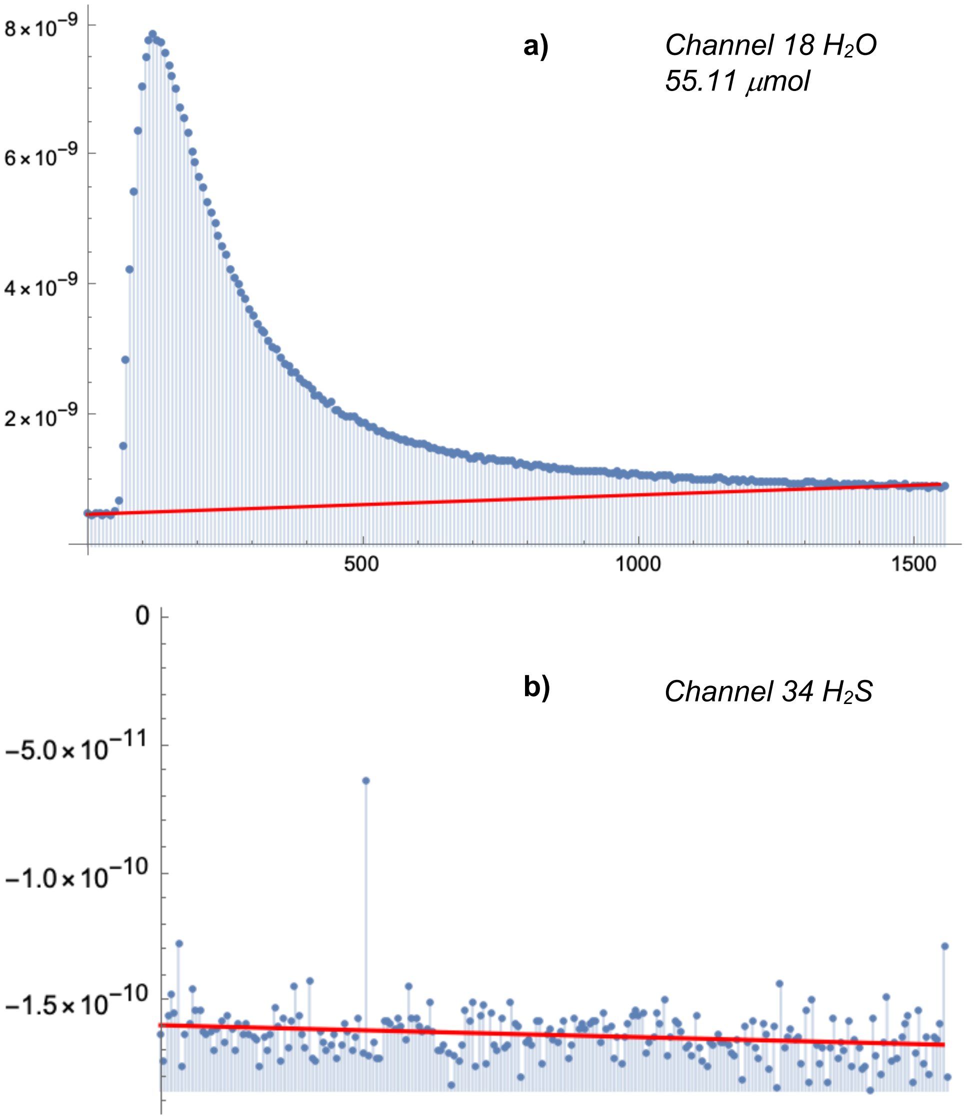

Supplementary Figure 4: Diagram reporting *m/z* signal integration (peak area) for **a)** channel 2 (H_2); **b)** channel 18 (H_2O); **c)** channel 34 (H_2S) and **d)** channel 64 (SO_2) measured during run SOH-IW5. Reported on x axis = time, on y axis = signal (a.u.)

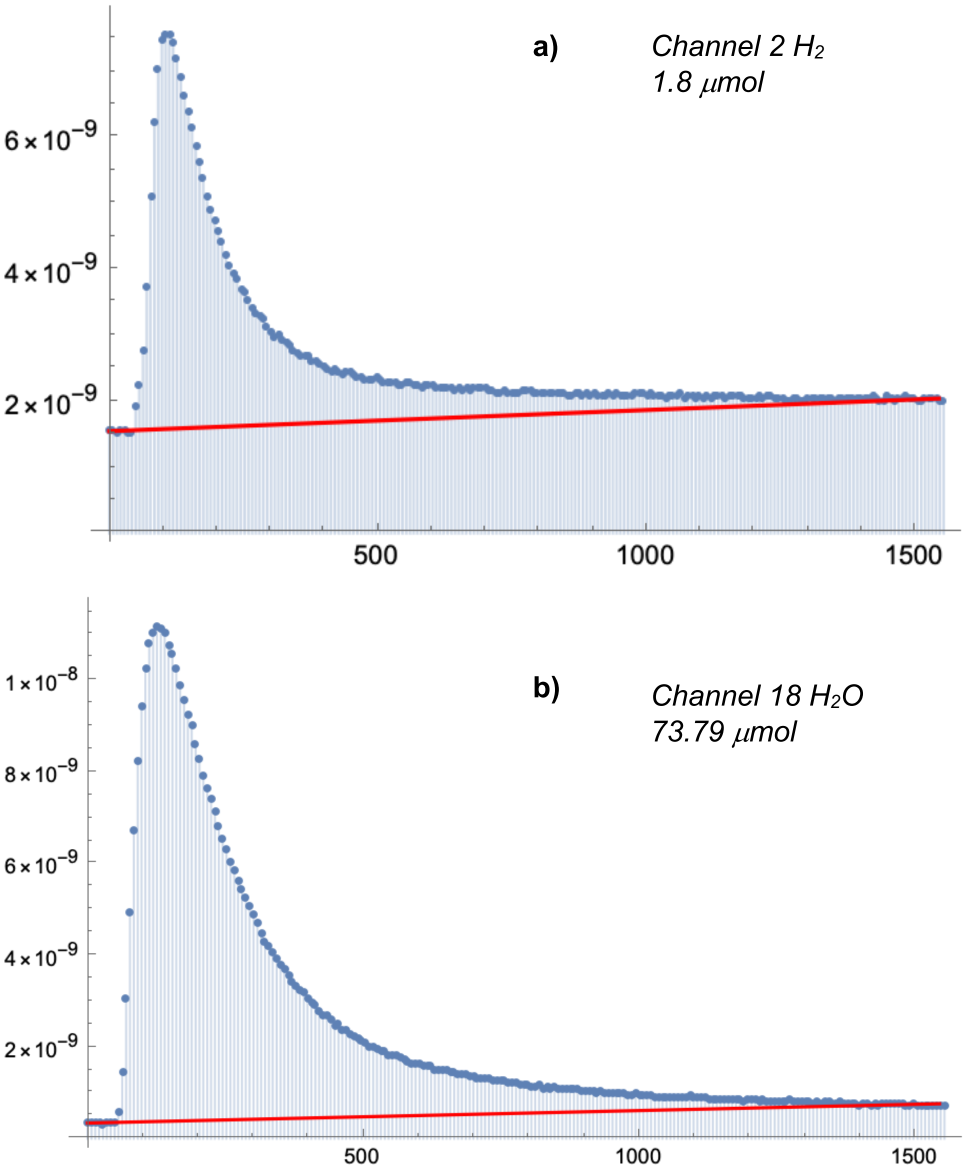

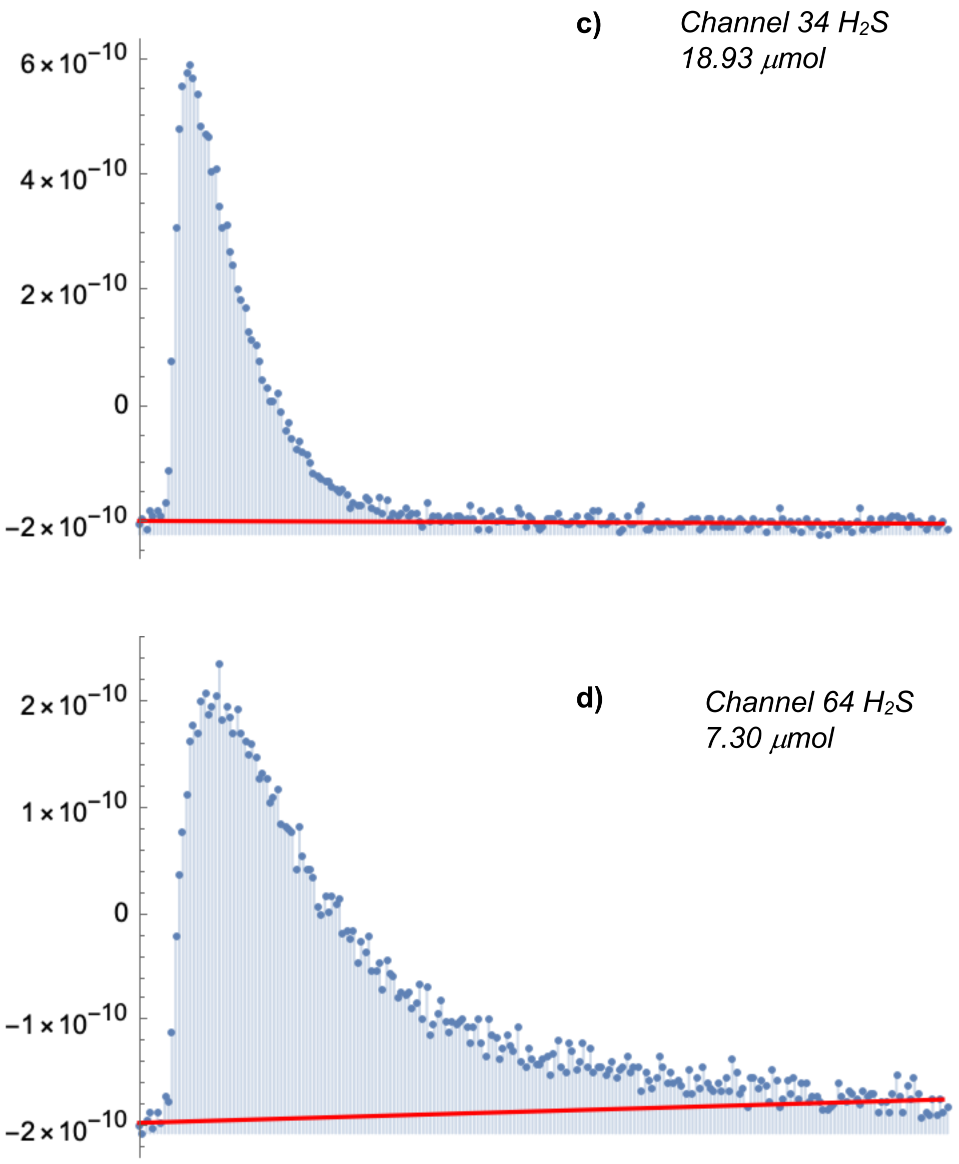

c)
Channel 34 H2S
18.93 μmol
6 × 10−10
4 × 10−10
2 × 10−10
0
−2 × 10−10
d)
Channel 64 H2S
7.30 μmol
2 × 10−10
1 × 10−10
0
−1 × 10−10
−2 × 10−10

Supplementary Methods

Au permeability test

Before establishing our protocol, we conducted a test experiment (SOH1, see Supplementary Table 1) to assess the permeability of the of the inner gold capsule to hydrogen at the relatively low temperatures explored in this study.

For this experiment, we have considered the C + H_2O system, known for being thermodynamically and experimentally[3] well-defined.

In the inner capsule, we placed a mixture of graphite and H_2O, while we the outer capsule contained FMQ as buffer assemblage. The experiment was run at conditions of 3 GPa and 700 °C, with a runtime of one week—matching the conditions of our SOH protocol. After the experiment, we analyzed the fluid composition and compared it with the equilibrium fluid composition predicted for 3 GPa, 700 °C and fH_2^{FMQ} using the Perple_X software package, using the thermodynamic dataset from Holland and Powell revised in 2004 (hp04ver.dat) and the graphite-saturated C–O–H hybrid MRK equation of state in the "fluids" routine. We also compared our results with previously published experimental data[3] obtained for fluid syntheses under identical P–T–fH_2 conditions (3 GPa, 700 °C, fH_2^{FMQ}) and comparable runtime (240 hours) using an Au–Pd double capsule, which is routinely used in experimental petrology for its well-known high hydrogen permeability. The results are reported below:

Run	P (GPa)	T (°C)	Runtime (h)	$X\,CO_2$ [a]	$X\,CO_2$ *liter* [b]	$X\,CO_2$ *thermod* [c]
SOH1	3	700	168	0.25	0.34	0.30

[a] $X\,CO_2 = CO_2/(H_2O+CO_2)_{molar}$ measured in experiment SOH1

[b] Experimental value from literature[3]

[c] Predicted value according to the G-COH thermodynamic model[3]

This experiment demonstrates that the inner gold capsule is permeable to hydrogen, confirming that a Au–Au double capsule system is a suitable setup for our protocol at the investigated *P–T* conditions.

Analysis of solids

Chemical analysis and back-scattered electron imaging were performed using a JEOL 8200 wavelength-dispersive electron microprobe at the Department of Earth Sciences, University of Milan, Italy. The running conditions were 15 kV acceleration voltage and 5 nA beam current, with a beam diameter of ~1 μm. A counting time of 30 s (10 s background) was applied for all the elements.

Image analysis was performed on X-rays elemental maps of sulfur and iron using a Wolfram Mathematica® routine.

Analysis of fluid species

To extract and analyze the volatile phase synthetized during the experiment and quenched at room temperature, the experimental capsules were mounted in a capsule-piercing device[4] connected to a quadrupole mass spectrometer (QMS). A detailed description of the capsule-piercing device and the QMS is provided below.

In addition, standardization for sulfur-bearing volatile species was performed using two gas mixtures of known composition:

1) Ar (99 vol%) + H_2S (1 vol.%), see Supplementary Fig. 5

2) Ar (99 vol%) + SO_2 (1 vol.%), see Supplementary Fig. 6

Unlike the gas mixtures used for carbon species[4], which typically contain the species of interest at 10% concentration, sulfur-bearing volatile species in the gas mixtures are less concentrated. This adjustment is necessitated by the aggressive nature of sulfur volatiles, especially H_2S, which could potentially damage the steel lines of the capsule-piercing system, if present in higher concentrations.

Capsule-piercing device

The capsule-piercing device consists of a PTFE reactor hosted in an electric furnace (Supplementary Fig 7). The capsule is placed in a sample holder integral with the hollow base of the reactor. As the base is screwed in, the capsule moves towards a milling cutter that eventually pierces it. Once punctured, the gas inside the capsule fills the reactor, which is maintained at approximately ~ 90 °C to ensure complete evaporation of water from the capsule. At the top of the reactor, two three-way valves (V3 and V4 in Supplementary Fig. 7) control the inlet for the Ar carrier gas and the outlet of the gas mixture to be analyzed by the mass spectrometer. The gas flows from the reactor through a stainless-steel piping to a three-way needle valve (V5), where a small flux is sampled by the spectrometer line. The majority of the gas is conveyed to another three-way valve (V6) that connects to a vacuum pump via the main line. The vacuum pump is primarily used for preliminary cleaning of the line and the reactor; during subsequent operations it is excluded from the line. The line segment

that goes from the reactor to the spectrometer is heated to 105 °C by a winded resistance to prevent water condensation. The carrier gas flux is regulated by a Bronkhorst® mass flow controller. The pressure in the line and in the reactor is measured by two high sensitivity transducers having an error of ± 1 mbar, while the temperatures of the line, reactor and furnace are provided by three K-type thermocouples. An Eurotherm nanodac™ PID recorder/controller enables monitoring of the temperatures and pressures and automatically performs temperature adjustments.

Quadrupole mass spectrometer

The composition of the volatile phase, i.e. the concentration of S volatile species in the fluid, was measured using an ExTorr Inc. XT200 quadrupole mass spectrometer (QMS) equipped with a secondary electron multiplier. The spectrometer operates under high vacuum (~5·10-7 Torr), which is obtained through a turbomolecular pump coupled with a backing pump (Edwards® T-Station 85). The QMS simultaneously measures 13 mass-to-charge (*m/z*) channels via a Faraday cup, covering the fragmentation products of key volatile species (H_2S, SO_2, H_2, H_2O, N_2, O_2), with data collection over 1554 s. Signal intensity is enhanced by employing a secondary electron multiplier during both background and sample acquisitions. Data are presented as time (s) versus partial pressure (Torr) elution profiles. Integration and conversion of these profiles to micromoles are performed using a dedicated Wolfram Mathematica® routine incorporating a calibration matrix. To address instrumental drift, the calibration is periodically updated using measurements of pure water, air, and specific gas mixtures:

1) Ar + H_2S (1 vol.%);
2) Ar + SO_2 (1 vol.%);
3) Ar + H_2 (10 vol.%).

Uncertainties in measurements are estimated at approximately 1 mol% for H_2O and H_2, while for H_2S we estimated a LOD of 0.21 µmol and a LOQ of 0.64 µmol.

Supplementary Figure 5: *m/z* peak analysis for the gas mixture used for H_2S determination: **a)** channel 34; **b)** channel 33.

Reported on x axis = time, on y axis = signal (a.u.)

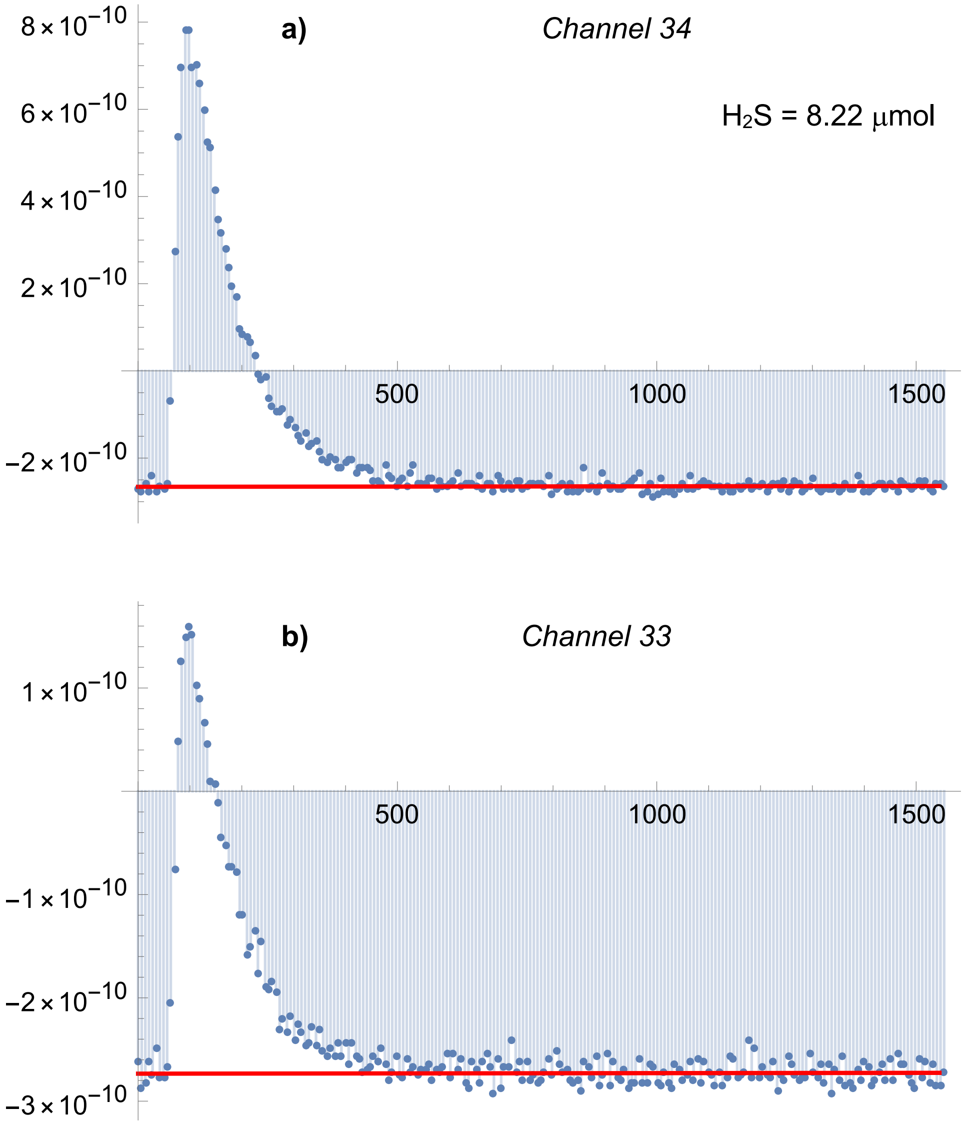

Supplementary Figure 6: *m/z* peak analysis for the gas mixture used for SO_2 determination: **a)** channel 64; **b)** channel 48.

Reported on x axis = time, on y axis = signal (a.u.)

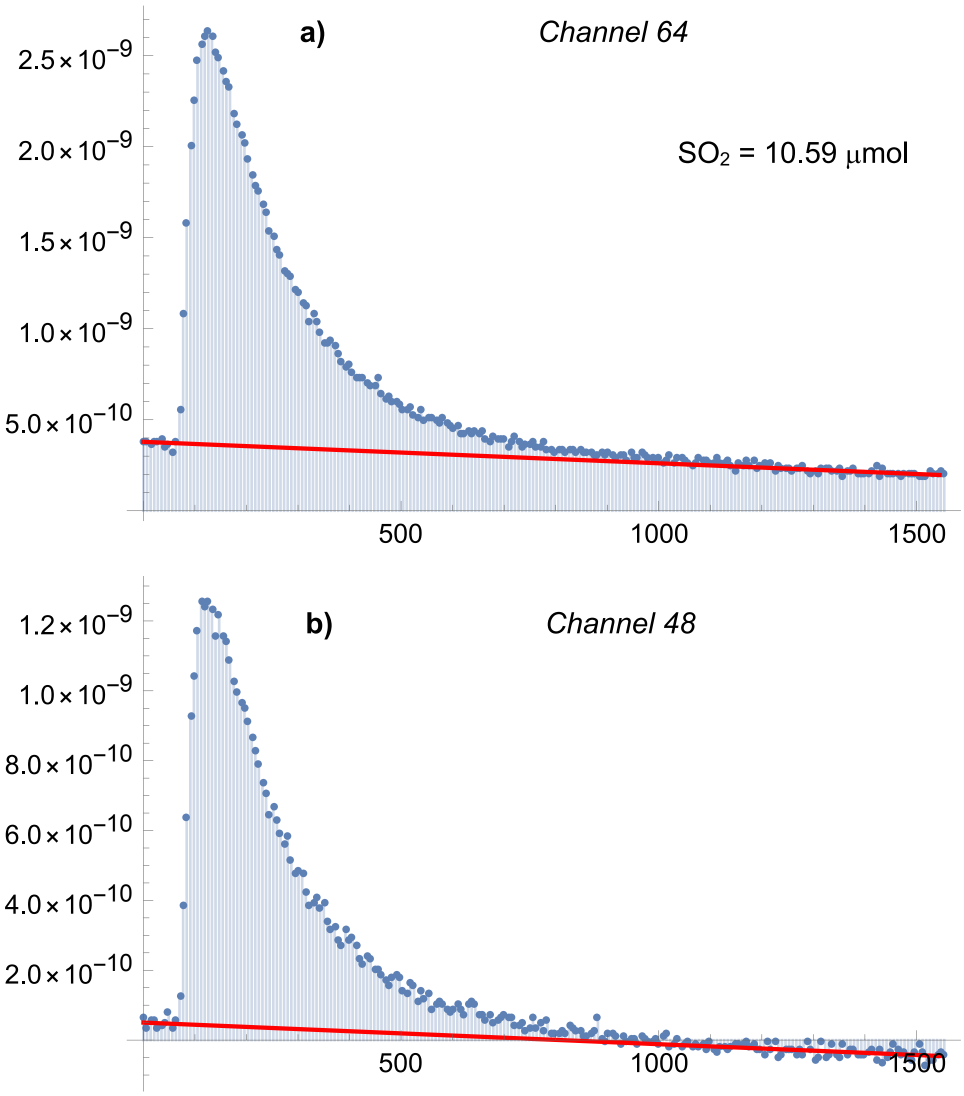

Supplementary Figure 7: layout of the line connecting the capsule piercing device with the QMS. MFC: mass flow controller; LPT: line pressure transducer; RPT: reactor pressure transducer.

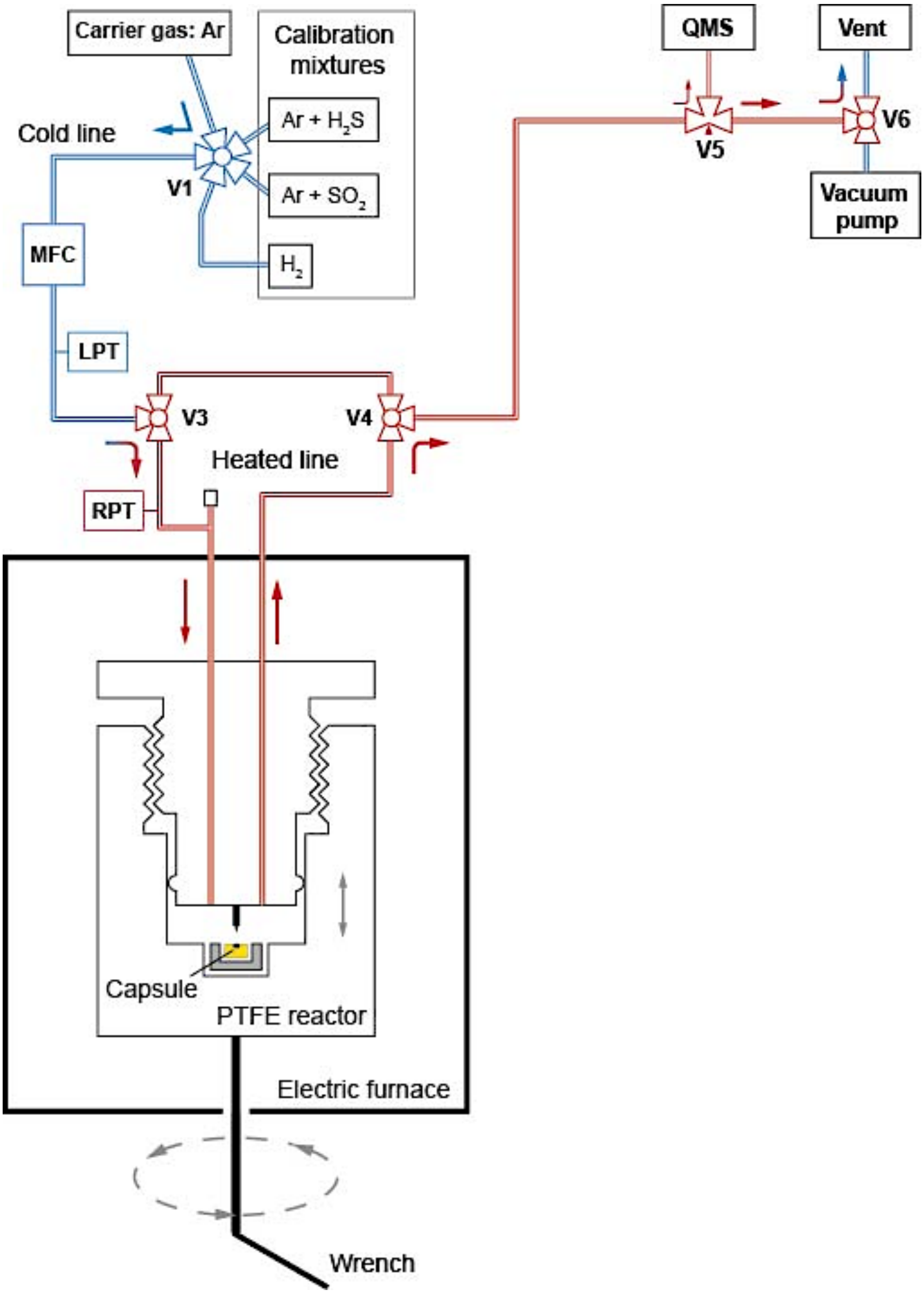

Supplementary Table 1

Run	Run name	Buffer	T (°C)	P (GPa)	Runtime (h)	Fluid	Note
SOH1	SOH-FMQ0	FMQ	700	3	168	Present	Permeability test ($C+H_2O$), fluid in equilibrium
SOH2	-	FMQ	700	3	168	Present	Fluid in equilibrium, not reported in this work
SOH3	-	IW	700	3	168	-	-
SOH5	-	IW	700	3	168	-	-
SOH6	-	IW	700	3	168	-	-
SOH10	-	IW	700	3	168	-	-
SOH11	-	IW	700	3	96	-	-
SOH12	-	IW	700	3	168	-	-
SOH13	-	IW	700	3	24	-	-
SOH14	SOH-IW1	IW	700	3	6	Present	Fluid not in equilibrium
SOH15	-	IW	700	3	24	-	-
SOH16	-	IW	700	3	12	-	-
SOH17	-	IW	700	3	7	-	-
SOH18	-	IW	700	3	10	-	-
SOH19	SOH-IW2	IW	700	3	5	Present	Fluid in equilibrium
SOH20	SOH-FMQ1	FMQ	700	3	5	Present	Fluid in equilibrium
SOH28	SOH-IW3	IW	800	3	5	-	-
SOH29	SOH-IW4	IW	600	3	5	Present	Fluid not in equilibrium
SOH30	SOH-IW5	IW	700	1	5	Present	-

Summary of the performed experiments

Supplementary Table 2

Synthesis	SOH-IW3	SOH-IW4	SOH-IW5
P (GPa)	3	3	1
T (°C)	800	600	700
Buffer assemblage	IW	IW	IW
Runtime (h)	5	5	5
µmol tot	Fluid not present	55.11	101.84
µmol			
H_2O	-	55.11 (0.05)	73.79 (0.35)
H_2	-	-	1.82 (0.04)
H_2S	-	-	18.93 (0.19)
SO_2	-	-	7.30 (0.31)
mol%			
H_2O	-	100.0	78.1
H_2	-	-	1.8
H_2S	-	-	18.6
SO_2	-	-	7.2

Additional experiments performed to evaluate the possible role of P and T on the capsule weakening effect and composition of the analyzed fluid. Volatile speciation of the SOH fluids synthethized under controlled P-T-redox conditions and measured by quadrupole mass spectrometry.
The total amount of fluid synthetized is expressed in mmol and calculated from the ideal gas law PV = nRT.
The amount of the monitored species (µmol) derived from linear regression analysis performed through Mathematica notebook. The volatile speciation of the SOH fluid is expressed as moles percentage on an air- and N_2-free basis (mol %).

Supplementary Table 3

Synthesis	SOH-IW1	SOH-IW2	SOH-IW2	SOH-FMQ1
Note	*average*	*core*	*rim*	*average*
Pyrite wt%	66.22	88.83	64.01	93.87
Pyrrhotite wt%	33.78	11.17	35.99	6.13

Results of the image analysis performed on the compositional maps of Fig. 3 and Supplementary Fig. 1 using Mathematica® routine.

Supplementary References

1. Syracuse, E. M. *et al.* The global range of subduction zone thermal models. *Physics of the Earth and Planetary Interiors* **183**, 73–90 (2010).
2. Sharp W.E. Melting curves of sphalerite, galena, and pyrrhotite and the decomposition curve of pyrite between 30 and 65 kilobars. *J Geophys Res* **74**, 1645–1652 (1969).
3. Tumiati, S. *et al.* Subducted organic matter buffered by marine carbonate rules the carbon isotopic signature of arc emissions. *Nat Commun* **13**, 2909 (2022).
4. Tiraboschi, C., Tumiati, S., Recchia, S., Miozzi, F. & Poli, S. Quantitative analysis of COH fluids synthesized at HP–HT conditions: an optimized methodology to measure volatiles in experimental capsules. *Geofluids* **16**, 841–855 (2016).
